# High resolution multibeam and hydrodynamic datasets of tidal channels and inlets of the Venice Lagoon

**DOI:** 10.1038/sdata.2017.121

**Published:** 2017-09-05

**Authors:** Fantina Madricardo, Federica Foglini, Aleksandra Kruss, Christian Ferrarin, Nicola Marco Pizzeghello, Chiara Murri, Monica Rossi, Marco Bajo, Debora Bellafiore, Elisabetta Campiani, Stefano Fogarin, Valentina Grande, Lukasz Janowski, Erica Keppel, Elisa Leidi, Giuliano Lorenzetti, Francesco Maicu, Vittorio Maselli, Alessandra Mercorella, Giacomo Montereale Gavazzi, Tiziano Minuzzo, Claudio Pellegrini, Antonio Petrizzo, Mariacristina Prampolini, Alessandro Remia, Federica Rizzetto, Marzia Rovere, Alessandro Sarretta, Marco Sigovini, Luigi Sinapi, Georg Umgiesser, Fabio Trincardi

**Affiliations:** 1 Consiglio Nazionale delle Ricerche, Istituto di Scienze Marine (CNR-ISMAR), Venezia, Castello 2737/f, Venice 30122, Italy; 2 Consiglio Nazionale delle Ricerche, Istituto di Scienze Marine (CNR-ISMAR), Bologna, Via Gobetti, 101, Bologna 40129, Italy; 3 Istituto Idrografico della Marina, Passo all’Osservatorio 4, Genova 16134, Italy; 4 Institute of Oceanography, University of Gdansk, al. Marszalka Pilsudskiego 46, Gdynia 81-378, Poland; 5 Smithsonian Environmental Research Center (SERC), 647 Contees Wharf Road, Edgewater, Maryland 21037, USA; 6 Department of Geology and Petroleum Geology, University of Aberdeen, King's College, Aberdeen AB24 3FX, Scotland; 7 Royal Belgian Institute of Natural Sciences, Operational Directorate Natural Environment Gulledelle 100, Brussels 1200, Belgium; 8 Renard Centre of Marine Geology, Department of Geology and Soil Science, Ghent University, Krijgslaan 281 s.8, B-9000 Gent, Belgium; 9 Open Access Centre for Marine Research, Klaipėda University, H. Manto 84, Klaipėda 92294, Lithuania

**Keywords:** Geography, Environmental chemistry, Hydrology, Physical oceanography

## Abstract

Tidal channels are crucial for the functioning of wetlands, though their morphological properties, which are relevant for seafloor habitats and flow, have been understudied so far. Here, we release a dataset composed of Digital Terrain Models (DTMs) extracted from a total of 2,500 linear kilometres of high-resolution multibeam echosounder (MBES) data collected in 2013 covering the entire network of tidal channels and inlets of the Venice Lagoon, Italy. The dataset comprises also the backscatter (BS) data, which reflect the acoustic properties of the seafloor, and the tidal current fields simulated by means of a high-resolution three-dimensional unstructured hydrodynamic model. The DTMs and the current fields help define how morphological and benthic properties of tidal channels are affected by the action of currents. These data are of potential broad interest not only to geomorphologists, oceanographers and ecologists studying the morphology, hydrodynamics, sediment transport and benthic habitats of tidal environments, but also to coastal engineers and stakeholders for cost-effective monitoring and sustainable management of this peculiar shallow coastal system.

## Background & Summary

Coastal transitional systems are amongst the most productive and valuable environments on Earth^1–4^. The hydrodynamics and related sediment, nutrient and biota exchange of these systems with the open sea is governed by their tidal networks, intricate patterns of bifurcating tidal channels dissecting tidal flats and salt marshes. Tidal networks are observed worldwide, with the best-known examples including the inlets of the East Coast of the United States, the Wadden Sea and the Lagoon of Venice. These environments often represent highly urbanized settings with half of the world’s population and 13 of the largest mega-cities located close to the coast. Therefore, tidal networks and coastal transitional environments undergo fast morphological changes under natural and anthropogenic pressures, which have lead to increased flooding and habitat losses (e.g., shrinking salt marshes) that are likely to further increase due to climate change^5–8^.

Despite being so relevant for the functioning of coastal transitional systems, tidal channels are still less studied than their river counterparts. The relatively few morphological observations on tidal channels rely mainly on limited-resolution 2-D topographic surveys of channel profiles and cross-sections like those in the Tijuana estuary^9^, and along the Schelt estuary in Belgium^10^, or on aerial or satellite images as for example in New Jersey^11^, and in Venice Lagoon^12,13^.

The complex three dimensional morphology of tidal channels is still poorly imaged because shallow transitional environments are difficult to map in a comprehensive way. Optical imaging of the bottom is very limited in turbid areas like these. Acoustic devices, including MBES, have had restricted use in depths of 2–5 metres, mostly due to side-lobe effect^14^, bottom reverberation or multiple reflections that were difficult to circumvent. Only the recent technological developments are enabling multibeam systems to achieve very high performances reaching resolutions up to 0.05 m and operating up to 1 m depths^8,15–17^.

**Figure 1:**
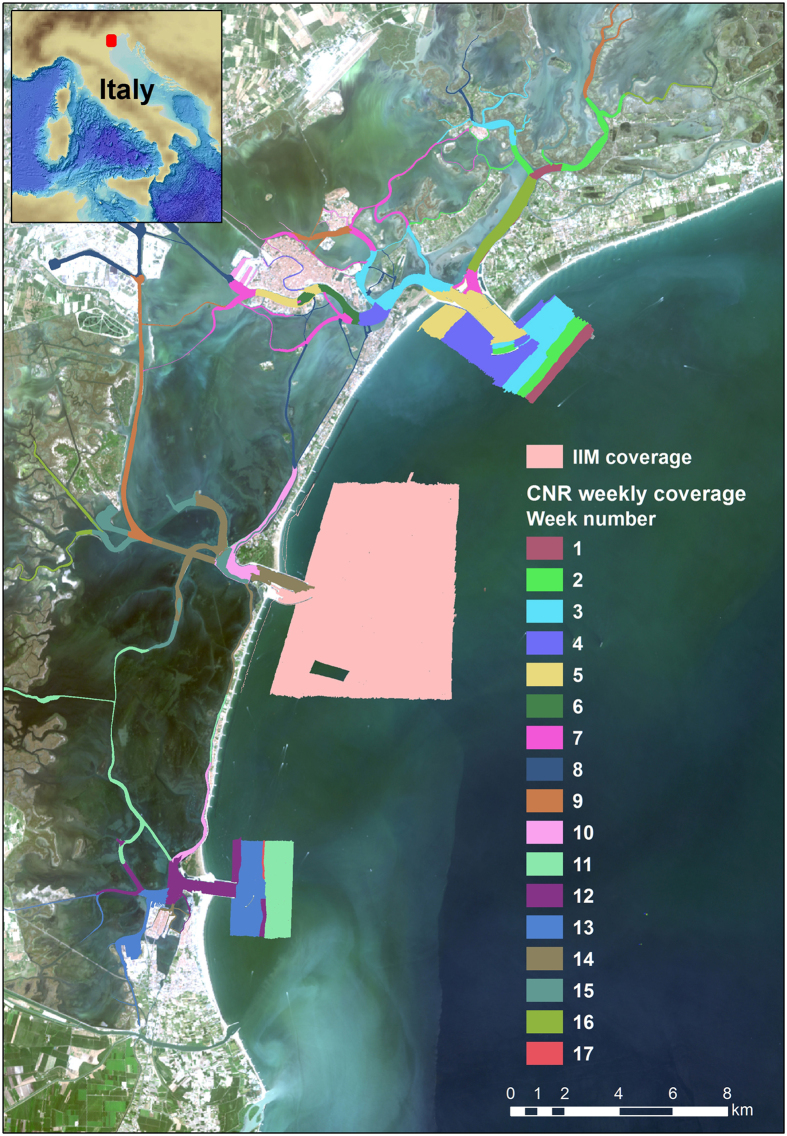
Working area with coloured polygons representing acquisition coverage by week numbers. The light pink polygon depicts the area surveyed by the *Istituto Idrografico della Marina* (IIM) (Italian Hydrographic Institute), whereas the coloured ones the CNR-ISMAR weekly covered areas. Pseudo-true-colour LANDSAT 8 OLI imagery as background.

The dataset presented in this paper contains high-resolution data collected in the channel network of the Venice Lagoon by means of a high-resolution, multi-frequency MBES (Fig. 1). The Venice Lagoon is the largest lagoon in the Mediterranean (550 km^2^ and with an average depth of about 1.5 m) and is one of the UNESCO World Cultural and Natural Heritage sites, including the historical city of Venice. Previous mapping of the whole Venice Lagoon were carried out in 1927, 1970 and 2002. Despite their low, and typically highly costly resolution (always >10 m), the resulting maps allowed a first recognition and semi-quantitative estimate of the erosional trends affecting the wetlands and of the deepening of the central lagoon^18^. In the last century, the lagoon morphology and ecological properties dramatically changed with a decrease of the salt marsh areas by more than 50% (shrinking from 68 km^2^ in 1927 to 32 km^2^ in 2002) and a net sediment flux modification^18–21^. Relative sea level rise will likely increase the frequency of flooding events in Venice^22–24^. To protect the city from floods (‘high water events’), a complex array of large mobile barriers is under construction (MOSE system) through major modifications of the three lagoon inlets. Once in full operational mode, the MOSE system might substantially affect the lagoon hydrodynamics and, consequently, sediment transport and sediment balance^25–27^. More generally, the MOSE system is a global engineering example for coastal protection against storm surges in a frame of overall mean sea level rise. A discussion on the implicit assumptions and limitations of the MOSE solution for the high water in Venice can be found in Trincardi *et al.*^24^.

Before the MOSE system begins to function, it was important to have a full picture of the bathymetry and currents of the tidal channels and inlets, which are the most dynamical portion of the lagoon. These data represent, therefore, a precise reference for monitoring and quantifying future modifications of the channel and inlets morphology and for analyzing trends of the lagoon future evolution.

**Figure 2:**
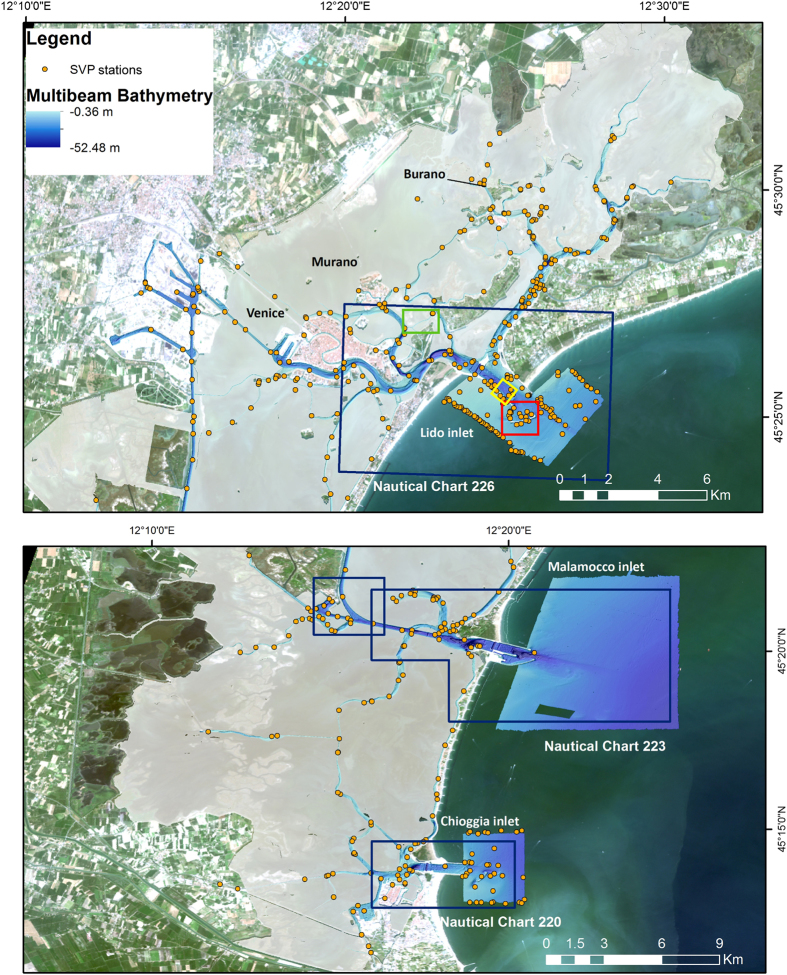
DTM from the multibeam bathymetry at 0.5 m resolution and 5 times vertical exaggeration showing the distribution of Sound Velocity Profiles (SVPs) collected during the survey. The blue boxes indicate the areas validated by the Italian Hydrographic Institute and the data included in the official nautical charts. The red, green and yellow boxes show the location of the areas in Figs 6–8, respectively, with pseudo-true-colour LANDSAT 8 OLI imagery as background.

In 2013, an extensive mapping was carried out by CNR-ISMAR within the project RITMARE (a National Research Programme funded by the Italian Ministry of Education, University and Research). A team of more than 25 scientists was involved to collect high-resolution bathymetry of the tidal channel network and the inlets (Figs 1 and 2). This survey merges with the complementary mapping of the lagoon inlets conducted by the Italian Hydrographic Institute in the offshore areas at the same time frame and using similar equipment.

Bathymetric and seafloor BS-data can now be employed for a variety of studies defining aspects of the evolution of the lagoon including: hydrodynamic modelling, sediment dynamics and geomorphology, geo-archaeology or habitat mapping. This dataset is unique not only because it depicts the seafloor morphologies with unprecedented detail but also, because it was acquired just before the MOSE barrier system starts to operate. Therefore it represents a benchmark for evaluating the possible impacts of the major engineering interventions taking place in the Lagoon of Venice and its inlets. The impact of hard structures on the seafloor is already visible outside the inlets where the high resolution bathymetry highlights the presence of large scours formed at the edges of semicircular breakwaters built between 2006 and 2011 as part of the MOSE project. The relative rapid erosive process could threaten the stability of the hard structures in the near future and should certainly be periodically monitored.

## Methods

### Multibeam data acquisition

The MBES raw data were collected during six-months (from May to December 2013) with a Kongsberg EM-2040 compact dual-head multi-frequency system. The survey areas, divided in 17 acquisition weeks, are not spatially contiguous because they were chosen depending on weather conditions and permit constraints. The MBES was pole-mounted on the CNR research vessel Litus, a 10-m-long boat with only 1.5-m-deep draft. The MBES has 800 beams (400 per swath) 1°×1°; the operational frequency for this survey was set at 360 kHz. A Seapath 300 system was used for ship positioning, supplied by a Fugro HP differential Global Positioning System (DGPS), accurate up to 0.2 m. The Kongsberg motion sensor MRU 5 and a Dual Antenna GPS integrated in the Seapath, corrected pitch, roll, heave and yaw movements (reaching 0.02° roll and pitch accuracy, and 0.075° heading accuracy). A Vale-port mini SVS sensor was attached close to the transducers to continuously measure the sound velocity for the beamforming. Sound velocity profiles (SVPs) were systematically collected (640 SVPs in total) with an AML oceanographic Smart-X sound velocity profiler (yellow dots in Fig. 2). Data were logged, displayed and checked in real-time by the Kongsberg data acquisition and control software SIS (Seafloor Information System). Professional topographers measured the offsets of the instruments with millimetric accuracy using a dedicated dimensional survey of the ship’s hull at dry dock.

Several error sources may influence the multibeam data, giving errors in both real time presentation and final product. To avoid these errors, sensors have been calibrated (roll, pitch, time and heading offsets) and regularly checked. A 40% overlap between lines was kept in order to avoid the influence of external beams of bad quality given by residual errors in roll and sound speed profile measurements. The dual-head multibeam operated with a swath opening angle of 70° for each head inside the channels and 60° in the inlets, maintaining 15° of overlap between the two swaths. The vessel sailed with a reasonably constant speed (4 knots mean speed), despite strong tidal currents (and heavy ship traffic).

### Multibeam data processing

#### Bathymetry

**Figure 3:**
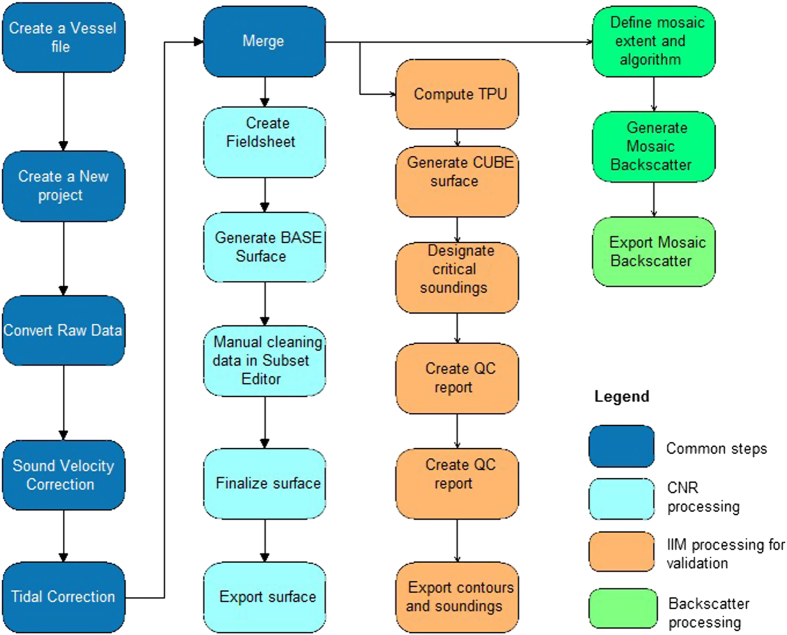
Workflow of the processing performed in CARIS for bathymetry and BS data.

**Figure 4:**
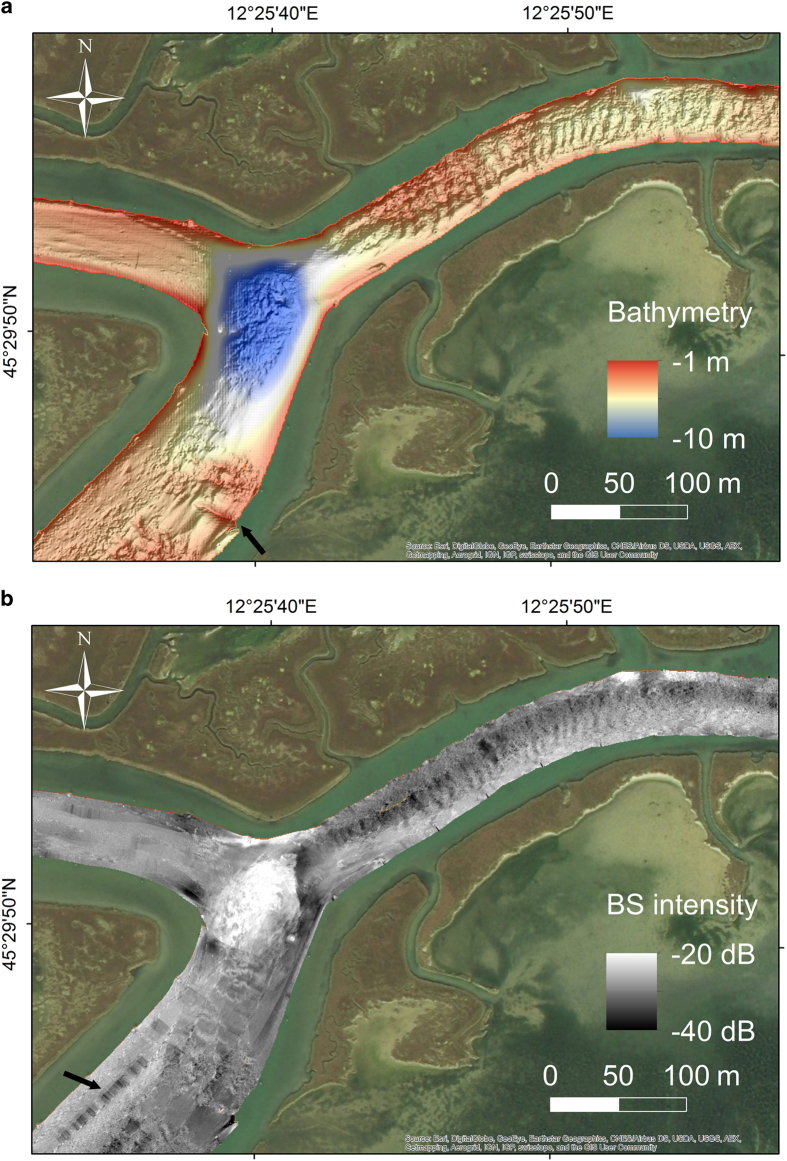
Example of multi beam data processing outputs. (**a**) DTM (0.5 m resolution and 5X vertical exaggeration) result of bathymetric data processing executed with CARIS HIPS and SIPS. The black arrow indicates the location of an alignment of wooden poles and amphorae dating back to Roman Times. (**b**) Example of BS-mosaic processed with CARIS HIPS and SIPS. The black arrow highlights the presence of artefacts.

CARIS HIPS and SIPS software (v.7 and 9.1)^28^ was used for processing multibeam data taking into account sound velocity profiles, tide corrections and manual quality control tools. The standard CARIS HIPS and SIPS workflow for bathymetry and BS data processing (Fig. 3) was followed. At the end of each week of acquisition at sea, a separate CARIS project was created, scoring totally 17 weeks of multibeam acquisition. The final combined surfaces (DTMs) with depths and BS are then divided into 17 parts referring to the acquisition week (Fig. 1). The bathymetry was created with the Swath Angles Weighting option with a Max Footprint size of 9×9 pixels and a resolution of 0.5 m and cleaned using the subset editor to handle and visualize efficiently the data (see Fig. 2 for the general DTM and Fig. 4 for an example of a DTM for bathymetry and BS mosaic).

**Figure 5:**
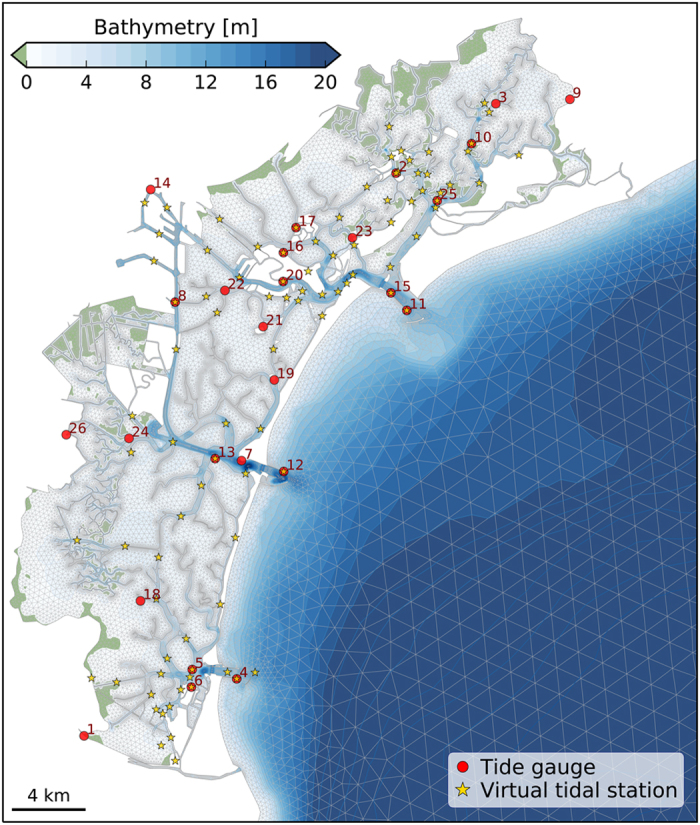
Unstructured numerical mesh of the hydrodynamic model SHYFEM with the bathymetry interpolated over the mesh elements. The red dots mark the location of the tide gauges (with ID according to Table 1) used in the model validation and the yellow stars indicate the location of the virtual tidal stations for the tidal correction procedure.

A set of 93 virtual tidal stations (see yellow stars in Fig. 5), evenly distributed in the study area, served for tidal correction: a virtual tidal station, was used for each field sheet of CARIS created for the data collected in a single day of survey. The tidal corrections in each virtual tidal station were calculated using the water level simulated by the hydrodynamic model SHYFEM^29,30^ applied to the whole Venice Lagoon with assimilation of tide gauge observations. All the corrections are referred to the local datum ‘Punta Salute 1897’. The Italian Hydrographic Institute reprocessed part of the bathymetric data (see Fig. 2) for the safety of navigation. Starting from the CARIS HIPS and SIPS projects, information about uncertainty of all experimental devices was computed into an uncertainty surface at 1-m-resolution, named Combined Uncertainty and Bathymetry Estimator (CUBE) surface. The CUBE surface provides multiple depth estimates for a single grid node, depending on the variation of the sounding data. The ‘Disambiguation’ tool was used to determine the best depth hypothesis at each node^31^ and to create the most accurate surface. This surface, completed with the information of horizontal and vertical propagated uncertainty, was vertically shifted to the mean low water spring datum and transferred into the database of the Italian Hydrographic Institute. The surface was then used for the official Italian nautical charts of the Lagoon^32–34^.

#### Backscatter Data

BS mosaics were created in CARIS HIPS and SIPS through the Geocoder engine. The Geocoder tool automatically corrects the system settings for transmission loss, insonification area and incidence angle variations^35^. Mosaics were generated using Angle Varying Gain (AVG) correction with a 300-pixel size window and Despeckle option in moderate mode to remove isolated pixels and improve the final intensity layer^28^. The same parameters were used for each mosaic generation. The bathymetric grids and BS mosaics were exported from CARIS with grid resolutions of 0.5 m as .txt and .geotiff files, respectively. The .txt files then were converted to 32-bit ESRI raster files using Global Mapper (v15). The raster files were imported into ArcGIS (v10.2)^36^ (ESRI 2015) for further analysis.

#### Hydrodynamic model

The hydrodynamic model SHYFEM developed by Umgiesser *et al.*^29,30^ was applied to correct the data for the tidal excursion and to simulate the water circulation of the lagoon. SHYFEM resolves the 3-D primitive equations vertically integrated over multiple *z*-layers and horizontally over an unstructured grid. The model is especially well suited to shallow-water areas. It has been successfully applied to the Venice Lagoon^29,27^ and to several other coastal systems^30^. The model uses a semi-implicit algorithm for integration in time. The spatial discretization of the unknowns is carried out with the finite element method, partially modified with respect to the classical formulation. The outcome is a grid that resembles a staggered grid often used in finite difference discretization.

The boundary conditions for stress terms (wind stress and bottom drag) follow the classic quadratic parameterization. Heat fluxes are computed at the water surface. Water fluxes between air and sea consist of the precipitation and runoff minus evaporation computed by the SHYFEM model.

Smagorinsky's formulation^37^ is used to parameterize the horizontal eddy viscosity. For the computation of the vertical viscosities, a turbulence closure scheme was used. This scheme is adapted from the k-*ϵ* module of GOTM (General Ocean Turbulence Model) described by Burchard and Petersen^38^. A detailed description of the 3-D model equations is given in Umgiesser *et al.*^30^.

#### Computation of the water levels and currents

The water circulation in the Venice Lagoon, induced by tide, wind, and water, heat and salt fluxes, was simulated by the unstructured model SHYFEM (described above, V7_1_4) applied over a spatial domain that represents the entire Lagoon and its adjacent shore. The model adequately reproduces the complex geometry and bathymetry of the Venice Lagoon using unstructured numerical meshes composed of triangular elements of variable form and size, going down to a few meters in the channels (Fig. 5). The water column is discretized into 22 vertical levels with progressively increasing thickness varying from 1 m for the upper 16 to 7 m for the deepest layer of the outer shelf. For the tidal excursion correction, the model bathymetry was obtained from the data collected in 2002 by *Magistrato alle Acque di Venezia* merged with later surveys. For the simulation of the tidal currents, the model integrated the 2002 dataset with the MBES bathymetry acquired by CNR-ISMAR in the main channels of the lagoon.

The model was run in a 3-D baroclinic mode using observed forcing and boundary conditions (i.e., wind stress, heat and salt fluxes, precipitation, sea level and freshwater discharge) provided by the City of Venice through the *Protezione Civile*—*Centro Previsioni e Segnalazioni Maree*, the Italian Institute for Environmental Protection and Research (ISPRA) and *Magistrato alle Acque di Venezia* (through THETIS spa).

The simulated sea surface elevation to correct MBES bathymetric data for tidal oscillations was obtained by assimilating into the state of the hydrodynamic model the hourly water levels recorded at the tide gauges marked with red dots in Fig. 5. Data assimilation was performed through a calibrated nudging scheme which considers each station to have a weight function based on an isotropic Gaussian spatial distribution and a constant relaxation time. The simulated water levels extracted in correspondence of the 93 virtual tidal stations (yellow stars in Fig. 5) were used in the tidal correction procedure.

Tidal currents have been computed by applying the model for both ebb and flood phases. The residual currents are obtained averaging the modelled currents over the one-year simulation (2014). Residual currents, induced by non-linear interactions between tides, wind and topography, in tidal embayment have been recognized as fundamental indicators of the net water movement and long-term sediment transport^39^.

### Code availability

The open source hydrodynamic model SHYFEM is freely available at the webpages www.ismar.cnr.it/shyfem and https://github.com/SHYFEM-model/shyfem.

## Data Records

The dataset of bathymetric data described in this paper is available on Marine Geoscience Data System (MGDS) (Data Citation 1). For the bathymetry a 0.5 m resolution DTM is provided as .*ascii* ESRI File format in 17 parts referring to date and time of the survey (divided into 17 weeks). For the BS data the mosaic for the most dynamical areas and the area surrounding the city of Venice (weeks 3 to 7, 9, 10, 16) is available in (Data Citation 2) at 0.5 m resolution as *geotiff* files. The raw bathymetric and BS data together with the SVP data are stored as. *all* and .*asvp* format in the Server, located at the CNR-ISMAR institute in Venice and are available upon request addressed to the first author of this paper. The raw data are archived following the acquisition date (survey week number and acquisition day). For each subset of data, a .*xls* table reports all the details about acquisition data that are useful for any data re-use or re-processing.

The water current dataset which includes flood, ebb and residual velocity fields are archived in a public repository (Data Citation 3). The following files are contained in the archive SHYFEM_venice.zip: 1) SHYFEM_venice_flood.nc (unstructured *netcdf* file with the u/v components of the 3D flood velocity defined on the mesh node); 2) SHYFEM_venice_ebb.nc (unstructured *netcdf* file with the u/v components of the 3D ebb velocity defined on the mesh node); SHYFEM_venice_residual.nc (unstructured *netcdf* file with the u/v components of the 3D residual velocity defined on the mesh node).

## Technical Validation

### MBES bathymetry data quality

A first general data quality assessment of the bathymetric data was carried out using IHO Standards for Hydrographic Surveys S-44, 2008. Total Propagated Uncertainty (TPU) was calculated in CARIS HIPS and SIPS 9^40^, considering installation offsets, MBES characteristics, position uncertainties and timing errors. This value results from the combination of all individual error sources. TPU can be split into horizontal (hzTPU) and vertical (dpTPU) components, related to the positioning of each sounding and depth-values uncertainties. The final uncertainties are scaled to S44 confidence level (95%) (Caris 2009). Mean values obtained for the whole dataset are hzTPU=0.4 m and dpTPU=0.1 m (referred to 20 m depth), and fall within the Special Order IHO minimum standard (IHO S N°44, 2008).

However, this assessment has some limitation since it refers to overall or average metrics. It gives better results for deeper waters and it may average out sharp transitions at the interface between two sections of survey, that in fact are present in some parts of the survey. Since the data were collected over an extended time frame (six months), they resulted as spatially ‘patchy’ with errors that could be related to the use of a discrete number of tidal stations for the tidal correction instead of a continuous real time kinematic system, that was not available at the time of the measurements. Anyway, the use of sea levels derived from a unique model application limits bathymetric artefacts that could emerge when using records of tide gauges having different reference datum.

**Figure 6:**
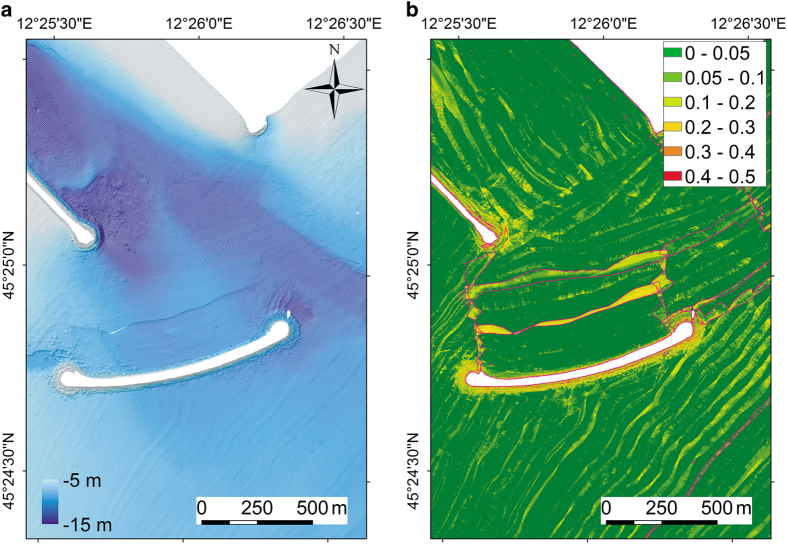
Example of visual evaluation of bathymetric data quality in the Lido Inlet. (**a**) Bathymetry and (**b**) standard deviation error in an area of the Lido Inlet (see Fig. 2) where the weeks 2–5 overlap. The pink polygons indicate the different week surveys.

By visually investigating all fieldsheets created at the junction areas between different weeks, we found that the difference at the edge of the week areas ranges from about 0.5 to 15 cm. This error is not linked to the absolute water depth, since we have the same vertical error in 5 m and 10 m average water depth. The maximum standard deviation was found in a very small area close to the Lido Inlet (Fig. 6). In this area the effect of the patchiness is maximum due not only to tidal correction but probably also to high variability of the sound velocity profiles. In the inlets, different water masses mix and due to temperature and salinity changes the sound velocity can vary a few meters per second in a short distance. We tried to limit this error increasing the density of sound velocity profiles sampling in the inlets (Fig. 2) and using sound velocity correction tool in CARIS HIPS and SIPS.

We also observed an error related to imprecise heave compensation that is due to the slow adjustment of the MRU system when the boat is turning more than 90° rapidly. Although, we tried to minimize this effect by waiting at the start of new lines after turning for MRU to stabilise, the error is still present in some parts of the survey at the beginning of survey lines. This error was quantified experimentally with repeated survey to be at most equal to 10 cm. Another error source can be related to the sound velocity and diffraction in the outer beams as it can be seen in Fig. 6. Finally, the presence of suspended sediment and submerged aquatic vegetation could potentially affect the MBES bottom detection and, locally, the resulting bathymetry.

### MBES BS data quality

In contrast to the bathymetric measurement, the BS data are the result of a complex interaction of the sonar echo with the water column and the seafloor. Therefore, more parameters need to be known or estimated to assess the error in BS data^41,42^:

1. The amplitude of the acoustic signal projected into the water that depends on the transmission power. In our case the transmission power was set up automatically during the survey depending on the water depth and the projector angular directivity pattern of the instrument. Both of these parameters are taken into account automatically during the mosaic creation in Geocoder engine;
2. The absorption and attenuation of acoustic energy that occurs in the travel of the acoustic wave through the water to the seafloor and back again. This energy loss depends on the signal-target range, on the physical properties of seawater (temperature and salinity versus depth) and on the signal frequency. During the experiments described below, we measured the influence of temperature and salinity fluctuations. We estimated that these factors could influence the results by no more than 0.5 dB.
3. The inherent uncertainty due to variations of the sonar receiver to acoustic signals that in our case is ±1 dB^43^;
4. Unwanted signal fluctuations. They were caused by the presence of air bubbles, schools of fishes and sediment suspension (less significant) in water column. All these elements can generate artifacts in the final BS mosaics. The bubbles influenced our data the most, reducing strongly the quality of BS data collected and, particularly, in the open sea outside the inlets of Lido and Chioggia. They were related either to turbulence in proximity of the transducers during surveys at rough sea or to presence of strong tidal currents, or of other boats navigating in the channels.
5. The influence of sediment suspension on surface BS data is still not fully understood. To estimate this effect, in the experiment described below, we used water column BS data, ADCP and turbidity measurements. The physical interaction of the pulse with the seafloor is influenced by seafloor roughness, hardness, angular dependence effects, and the presence of a biogenic substrate.
6. The corrections applied by the processing software.

**Figure 7:**
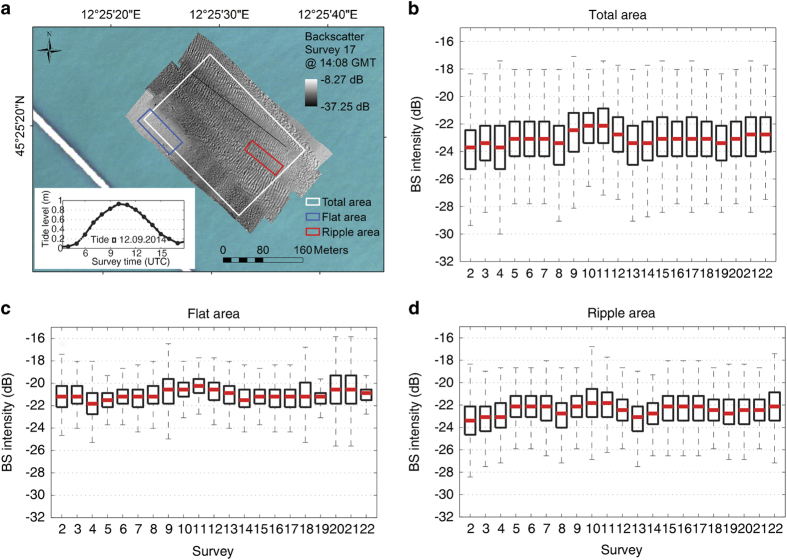
Variability of seafloor BS intensity over a tidal cycle in different areas of the Lido inlet. (**a**) Map of the BS collected during one of the surveys of Experiment 1 in the Lido Inlet and values of the tide during the experiment; (**b**) boxplots extracted from the mosaics of the total surveyed area (white polygon); (**c**) of a flat area (blue polygon) and (**d**)of a ripple area (red polygon), respectively.

**Figure 8:**
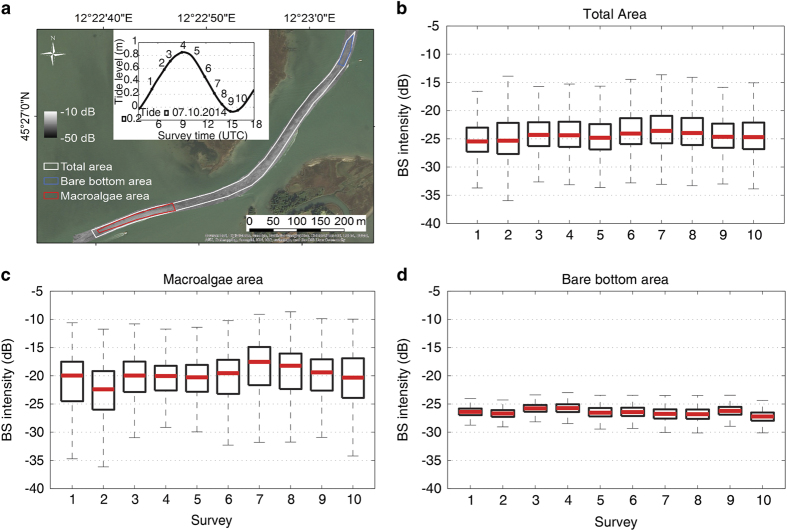
Variability of seafloor BS intensity over a tidal cycle in the La Bissa channel. (**a**) Map of the BS collected during one of the surveys of Experiment 2 in the La Bissa channel and values of the tide during the experiment; (**b**) boxplots extracted from the mosaics of the total surveyed area (white polygon); (**c**) of the bare bottom area (blue polygon) and (**d**)of the macroalgae area (red polygon), respectively.

In order to assess the global fluctuation of the BS data related to these factors, two experiments were carried out with repeated MBES surveys in different parts of the lagoon: a) in the Lido Inlet^44^ (Fig. 7); b) in a very shallow navigation channel (La Bissa Channel) covered by submerged aquatic vegetation (SAV)^45^(Fig. 8).

The experiment of the Lido Inlet was undertaken on the 12th September, 2014 between 0700 (GMT) and 1,700 (GMT) in a region of migrating megaripple fields (Fig. 7). A total of 22 repeated surveys were carried out with the MBES frequency set to 340 kHz and with more than 30% overlap between survey lines.

The BS intensity distribution was extracted from each mosaic created from the MBES data following the workflow described before. We elaborated the data for the full area covered by all surveys (white polygon in Fig. 7) and for two subsets extracted in a flat area (blue polygon in Fig. 7) and a ripple area (red polygon in Fig. 7). We excluded the first survey from the analysis since its coverage area was too small. By comparing the distributions in the boxplots, we found that the variability of the average value of the BS intensity extracted by the mosaics falls within the Kongsberg EM2040 DC instrumental sensitivity range (±1 dB).

During the experiment, we measured also concentrations of suspended sediment using turbidity meter, ADCP and Niskin bottles for calibration. We did not find any correlation between these concentrations variability over a time (range: 3–18 mg/l) and the surface BS.

The experiment in the La Bissa Channel (described in detail in Kruss *et al.*^45^) was a 12 h experiment with 10 repeated surveys over a tidal cycle, one about every hour with 320 kHz of frequency. To estimate the variability of the seafloor BS intensity, also in this case, we show the BS intensity distribution over time as boxplots.

The analysis was carried out for the mosaics of the full area and two testing areas of the channel. These areas were representative of two seafloor types: a) a bare bottom area; b) an area covered by macroalgae (Fig. 8). They were selected on the basis of dropframe video data collected on the experiment day. This analysis showed that there were no significant changes of the BS for the full area and the bare bottom area with variations of the median value falling within the interval of instrumental sensitivity. For the macroalgae area, instead, the median values of survey 2 and survey 7 differ by about 6 dB, well outside the instrumental sensitivity range. This is due to the fact that the data were collected during ebb and flood tide, respectively. The strong tidal currents influenced the distribution of the macroalgae and their density and orientation. This BS fluctuation is therefore related to a real change in the substrate properties.

### SHYFEM water level data quality

The application of the SHYFEM model to the Venice Lagoon has been validated in previous works reproducing correctly the tidal propagation, the water flows in the three inlets and both the water temperature and salinity patterns^29^. Moreover, because the variations of sea level are of utmost importance for the correction of MBES data, extensive validation of numerical simulations against tide gauge data was performed. Through an iterative optimization procedure over a two-week hydrodynamic simulation, we calibrated the relaxation time (100 s) and the sigma distance of the Gaussian function (2,500 m) for the nudging assimilation scheme. Afterward, the hydrodynamic model was validated by comparing the numerical results with sea levels recorded in the lagoon (red dots in Fig. 5). The validation was performed by running 25 simulations of one month period in which the model assimilated the sea level data of all tide gauge stations except one station. For each simulation, the water levels computed in correspondence of the non-assimilated station were used to evaluate the model performance in reproducing the water level over the lagoon.

The model results compare reasonably well with the measurements with an overall root mean square error (RMSE) of 2.1 cm and a correlation coefficient (R^2^) close to 1 in all stations (Table 1). The RMSE generally increases following the tidal propagation from the inlet to the inner lagoon, with the highest differences found in the distal parts of the lagoon (stations 1, 3, 9, 14, 18, 26). The difference between the mean of model results and the mean of observations varies in the range of±2 cm without a clear pattern. It has to be noted that the local tide gauges are affected by uncertainty in the vertical reference level. Therefore, the use of modelled sea level guarantees a uniform reference level throughout the lagoon basin.

## Additional Information

**How to cite this article:** Madricardo, F. *et al.* High resolution multibeam and hydrodynamic datasets of tidal channels and inlets of the Venice Lagoon. *Sci. Data* 4:170121 doi: 10.1038/sdata.2017.121 (2017).

**Publisher’s note:** Springer Nature remains neutral with regard to jurisdictional claims in published maps and institutional affiliations.

## Supplementary Material



## Figures and Tables

**Table 1:** Statistical analysis of simulated water levels

**ID**	**Station Name**	**RMSE**	**R**^**2**^	**BIAS**
1	Botte Trezze	4.34	0.97	−1.40
2	Burano	2.02	0.99	0.01
3	Canal Ancora	3.13	0.99	−0.35
4	Chioggia Diga Sud	1.39	1.00	−0.14
5	Chioggia Porto	1.56	1.00	0.17
6	Chioggia Vigo	1.64	1.00	0.66
7	Faro Rocchetta	1.14	0.99	−1.45
8	Fusina	2.14	1.00	1.72
9	Grassabó	4.21	0.97	−0.55
10	Le Saline	2.42	0.99	−0.05
11	Lido Diga Sud	1.42	1.00	−0.70
12	Malamocco Diga Nord	1.25	0.99	−1.91
13	Malamocco Porto	1.38	1.00	−0.22
14	Marghera	2.65	0.99	0.00
15	Meda Bocca Lido	1.59	0.99	−0.96
16	Misericordia	1.94	1.00	0.09
17	Murano	1.86	1.00	0.55
18	Petta de Bo	3.70	0.99	1.12
19	Poveglia	1.90	1.00	0.06
20	Punta della Salute	1.61	1.00	0.14
21	Sacca Sessola	1.81	1.00	0.24
22	San Giorgio in Alga	1.73	1.00	0.45
23	Sant Erasmo	1.81	0.99	−0.11
24	Torson di Sotto	2.00	1.00	1.06
25	Treporti	1.74	0.99	0.47
26	Valle Averto	3.36	0.98	0.90
	Average	2.14	0.99	−0.01
Analysis results are given as centered root mean square error (RMSE), correlation coefficient (R^2^) and difference between mean of model results and mean of observations (BIAS). Unit is cm.				

## References

[d1] Marine Geosciences Data System Madricardo F. Foglini F. Trincardi F. 2016 http://dx.doi.org/10.1594/IEDA/323605

[d2] Marine Geosciences Data System Madricardo F. Foglini F. Trincardi F. 2016 http://dx.doi.org/10.1594/IEDA/323853

[d3] PANGAEA Ferrarin C. Umgiesser G. 2016 https://doi.org/10.1594/PANGAEA.867918

